# Transcriptional Profiling of a Patient-Matched Cohort of Glioblastoma (IDH-Wildtype) for Therapeutic Target and Repurposing Drug Identification

**DOI:** 10.3390/biomedicines11041219

**Published:** 2023-04-19

**Authors:** Aideen C. Roddy, Caitríona E. McInerney, Tom Flannery, Estelle G. Healy, James P. Stewart, Veronica J. Spence, Jamie Walsh, Manuel Salto-Tellez, Darragh G. McArt, Kevin M. Prise

**Affiliations:** 1 Patrick G. Johnson Centre for Cancer Research, Queen’s University Belfast, Belfast BT9 7AE, UK; 2 Department of Neurosurgery, Royal Victoria Hospital, Belfast Health and Social Care Trust, Belfast BT12 6BA, UK; 3 Regional Service for Neuropathology, Institute of Pathology, Royal Victoria Hospital, Belfast Health and Social Care Trust, Belfast BT12 6BA, UK; 4 Integrated Pathology Unit, Division of Molecular Pathology, The Institute of Cancer Research, Sutton SM2 5NG, UK

**Keywords:** brain tumor, recurrent glioblastoma, patient-matched samples, whole transcriptome profiling, Clariom^TM^ D Human Assay, connectivity mapping, gene signatures, drug repurposing, longitudinal study, QUADrATiC software

## Abstract

Glioblastoma (GBM) is the most prevalent and aggressive adult brain tumor. Despite multi-modal therapies, GBM recurs, and patients have poor survival (~14 months). Resistance to therapy may originate from a subpopulation of tumor cells identified as glioma-stem cells (GSC), and new treatments are urgently needed to target these. The biology underpinning GBM recurrence was investigated using whole transcriptome profiling of patient-matched initial and recurrent GBM (recGBM). Differential expression analysis identified 147 significant probes. In total, 24 genes were validated using expression data from four public cohorts and the literature. Functional analyses revealed that transcriptional changes to recGBM were dominated by angiogenesis and immune-related processes. The role of MHC class II proteins in antigen presentation and the differentiation, proliferation, and infiltration of immune cells was enriched. These results suggest recGBM would benefit from immunotherapies. The altered gene signature was further analyzed in a connectivity mapping analysis with QUADrATiC software to identify FDA-approved repurposing drugs. Top-ranking target compounds that may be effective against GSC and GBM recurrence were rosiglitazone, nizatidine, pantoprazole, and tolmetin. Our translational bioinformatics pipeline provides an approach to identify target compounds for repurposing that may add clinical benefit in addition to standard therapies against resistant cancers such as GBM.

## 1. Introduction

Glioblastoma, IDH-wildtype (GBM), is the most aggressive malignant Grade IV brain tumor occurring in adulthood [1]. GBM were formerly designated as either IDH-wildtype or IDH-mutant; however, the latter subtype has since been re-classified by the World Health Organization (WHO) as Grade IV astrocytoma IDH-mutant [2]. GBM are highly heterogeneous tumors, both histologically and clinically. Outcomes for patients remain poor, with a median overall survival of ∼14 months [3] and a 5-year survival rate of only 5% [4]. GBM are characteristically infiltrative with invasive edges that preclude complete surgical resection. Despite radical resection followed by the current standard-of-care delivered as fractionated radiation and concurrent temozolomide (TMZ) therapy (“Stupp protocol”), local relapse is inevitable [5]. Resistance to chemoradiotherapy is thought to be due to several factors, including the presence of GBM stem-like cells (GSC), which may be key in mediating tumor relapse [6,7]. Indeed, this tumor sub-population possesses enhanced invasiveness, DNA repair, and drug-efflux pumps that mediate evasion and resistance to current surgical, chemo, and radiation therapies, driving tumor relapse [8]. The conversion of non-GSC to GSC in tumors may be induced by microenvironment conditions such as hypoxia [9] and drugs such as TMZ [10].

Longitudinal profiling of GBM can provide insight into gliomagenesis, intratumoral heterogeneity, tumor evolution, as well as therapy resistance mechanisms [11,12,13,14]. Relatively few longitudinal studies have been carried out for recurrent GBM (recGBM) because tumors are often necrotic and ~70% are inoperable [15]. Therapeutic options for recGBM are currently dictated by the test results of molecular markers assessed from the initial precursor brain tumor that provide the integrated final diagnosis based on contemporaneous WHO classification guidelines [2]. Molecular alterations assessed in brain tumors include IDH1/2 mutations, Histone H3F3A mutation, MGMT promoter methylation, TERT promoter methylation, EGFR amplification, ATRX retention or loss, and 1p/19q chromosomal co-deletion, for example. Increasing evidence shows that this provides an inaccurate representation of the key biological mechanisms active in recGBM. Thus, it is essential to observe transcriptional changes occurring between initial and recurrent tumors to understand the changing biology to enable clinicians to better direct treatment strategies. To this end, several studies have assessed longitudinal transcription profiles of progressing GBM [16,17,18]. This has revealed multiple mechanisms of therapeutic resistance to the standard treatment protocol for initial tumors by studying expression changes between matched initial and recurrent tumors [16]. Another study, which used spatial sampling, revealed all three cellular phenotypes (neuronal, mesenchymal, proliferative) were present in recurrent IDH-wildtype gliomas, highlighting the transcriptional variability of GSC that contribute to therapy resistance [17]. Targeted therapies are urgently required for GBM, and several natural products and their chemical derivatives are being tested as therapeutic strategies in GSC [19]. Drug repurposing may offer an alternative strategy for identifying new GBM-targeted therapies that are already approved for clinical use.

Gene expression connectivity mapping is a method used to identify potential therapeutic compounds for drug repurposing [20]. The method involves a systematic approach to identify functional connections between gene expression signatures associated with biological phenotypes (e.g., physiological processes and diseases) with the mechanisms of action of bioactive compounds or drugs contained within the Library of Integrated Network-based Cellular Signatures (LINCS). Our group developed a novel standalone connectivity mapping software called QUADrATiC (QUB Accelerated Drug and Transcriptomic Connectivity) [21]. This software uses a statistical ranking algorithm to identify connections between a query gene list and a database of reference profiles. Profiles have been obtained by applying a variety of perturbagens or treatments to a range of cell lines and quantifying the resulting transcriptional changes. QUADrATiC uses, as its reference, a subset of the LINCS dataset, which is limited to those small-molecule compounds which have FDA approval and allows this to be analyzed to produce a list of statistically significant connections to a query gene list.

This study characterizes the transcriptional changes occurring between initial and recurrent GBM IDH-wildtype using patient-matched samples and a whole transcriptome approach. A differential gene expression analysis of the cohort returned a list of recurrence-specific genes. These were validated using evidence from the literature and four public expression datasets [4,16,22,23]. Functional analyses revealed that angiogenesis and immune-related processes were potentially up-regulated in recGBM tumors. The gene signature was further analyzed in the in silico drug screen software, QUADrATiC [21]. FDA-approved drugs that could potentially reverse or target the transcriptional signaling associated with the recGBM phenotype were identified. These novel candidate compounds may add clinical benefit in combination with the current standard therapy to prevent GBM recurrence.

## 2. Materials and Methods

### 2.1. Sample Collection and Molecular Profiling of GBM IDH-Wildtype Tumors

A cohort of 25 GBM patients was identified, and informed consent (ORECNI 10/NIR01/13) was obtained by the neurosurgeon (T.F.) prior to surgical resection. Samples were collected during surgery at the Royal Victoria Hospital, Belfast. Tumors were formalin-fixed paraffin-embedded and prepared as hematoxylin and eosin-stained slides. Histological diagnosis was confirmed by the neuropathologist (E.G.H.). Samples were archived at the Northern Ireland Biobank (NI Biobank; Project Ref NIB16-0218), which is a Human Tissue Authority Licensed Research Tissue Bank with generic ethical approval from The Office of Research Ethics Committees Northern Ireland (ORECNI REF 21/NI/0019) to confer ethical approval for projects (subject to application). A total of 15 patients were eligible to be included in the study based on the following criteria: (1) the patient initially presented with primary GBM, which subsequently recurred after undergoing a course of Stupp protocol; (2) tissue samples from both tumors (initial, recurrent) were retained in the NI Biobank; (3) tumors were immunostained (with primary antibodies to ATRX, IDH1 (R132) and MIB1) and molecularly profiled at the Molecular Neuropathology Laboratory, University College London. All information was interpreted to provide an integrated final diagnosis for tumors based on contemporaneous WHO classification guidelines [2,24].

### 2.2. RNA Extraction, Microarray Profiling, and Data Quality Control

Samples were provided by NI Biobank. In brief, total RNA was extracted from macrodissected tissue and amplified using the GeneChip WT Pico Reagent Kit (Thermo Fisher Scientific, Wilmington, NC, USA). The biotinylated sense-stranded DNA was hybridized to the Clariom^TM^ D Human array (Thermo Fisher Scientific, Wilmington, NC, USA) and profiled. Transcriptome Analysis Console (TAC; Thermo Fisher Scientific) software was used to conduct quality control (QC) assessments and data summarization prior to further analysis (see Supplementary Materials for details).

### 2.3. Provisional Comparisons of Initial and Recurrent GBM

Transcriptional profiles of initial and recurrent tumors were compared using several methods. A principle component analysis (PCA) was carried out using TAC software. Tumor purity was assessed using a data subset with the ESTIMATE R package [25] and statistically compared between groups using a *t*-test in SPSS (IBM). Transcriptional subtyping was carried out on all samples using the ssGSEA classification method [23]. Results for Proneural, Mesenchymal, and Classic subtypes were compared between initial and recurrent samples (see Supplementary Materials for more details).

### 2.4. Differential Gene Expression Analysis between Initial and Recurrent GBM

A differential gene expression analysis was implemented to compare initial and recurrent GBM using the Bayes correction method and a repeated-measure ANOVA with TAC software. This method pushes gene-wise residual variances towards a global trend, thereby improving statistical power, which is optimal for small sample sizes. In addition, this method automatically adjusts for multiple testing by assuming that 1% of the probes or genes are expected to be differentially expressed (see Manual). Results were filtered using *p*-value (<0.05) and expression fold change (<−2 or >2) threshold cut-offs. Differentially expressed probes or genes (DEGs) were visualized as a volcano plot and categorized into different functional groups.

The expression patterns of DEGs were further examined in initial and recurrent GBM using semi-supervised hierarchical clustering analyzed with TAC software. Euclidean distance was applied to measure the distance between objects, and the complete linkage method was used to measure the distance between clusters. Heatmaps were visualized for all DEGs and for only those which mapped to known annotations in the Ingenuity knowledge base from IPA Software (IPA®, QIAGEN Redwood City, USA).

To validate DEGs, expression patterns for the genes were compared between initial and recurrent GBM in four public cohorts. This included the Chinese Glioma Genome Atlas (CGGA), which according to the clinical data, its patients had similarly been treated with radiotherapy (77.5%) and chemotherapy (81%) [22]. Expression data available for DEGs were compared between initial (*n* = 85) and recurrent (*n* = 75) GBM IDH-wildtype from CGGA using a paired *t*-test in R. In addition, expression data available for DEGs were further compared between patient-matched initial and recurrent GBM from the Wang et al. study [23]. This included datasets from Kwon et al. (N = 15) [16], TCGA-GBM (N = 13) [4] and HF-MDA (Henry Ford Hospital—processed at MD Anderson; N = 9) [23]. Statistical comparisons were implemented with a paired *t*-test using the RecuR web portal (http://recur.bioinfo.cnio.es/ (accessed on 1 October 2022)). For each comparison, a threshold cut-off of *p*-value < 0.05 was applied to determine the statistical significance of the test.

Further validation of DEGs was sought through the scientific literature using a PubMed search. For each gene name, the search terms ‘glioblastoma’ and ‘GBM’ were included both with and without the additional prefix term ‘recurrent’. Results in scientific articles were examined to determine the directionality of expression of the DEGs in recGBM and the methodology used for data collection.

### 2.5. Functional Enrichment Analyses

#### 2.5.1. Gene Ontology Enrichment Analysis of the DEGs

DEGs were analyzed in a Gene Ontology (GO) enrichment analysis with Panther software using a web portal (http://geneontology.org/, accessed on 1 October 2022) [26]. All probes that were expressed in samples (N = 14,893) were exported from TAC software and used as a background set for the analysis. The DEG list was uploaded, and genes that were uniquely mapped were used for the GO analysis. Genes that are over- or under-represented in gene lists from GO terms are identified by comparing the background frequency to the sample frequency using a Fisher’s exact test. The background frequency is the number of genes annotated to a GO term in the entire background set, while sample frequency is the number of genes annotated to that GO term in the input list of DEGs. The statistical test result *p*-value is then the probability or chance of seeing at least x number of genes out of the total n genes in the list annotated to a particular GO term, given the proportion of genes in the whole genome that are annotated to that GO term. GO terms are then categorized based on their function as either biological process, molecular function, or cellular component. Results were corrected for multiple testing using FDR and adjusted *p*-values. The top ten results from each category and the number of genes enriched in that pathway were plotted using the ggplot2 package in R.

#### 2.5.2. Canonical Pathway Analysis

DEGs were analyzed in a canonical pathway analysis using IPA software. The DEG list generated in TAC software was uploaded as a new dataset, and mapped identifiers were used in a core analysis with default settings for significance (−log(*p*-value) > 2). Pathways with a z-score of greater or less than ±1.5 were plotted to reveal predicted activation or inhibition in the recGBM group. Results identify relevant relationships, pathways, mechanisms, and functions given a differentially expressed gene list and their corresponding *p*-values and expression fold change.

### 2.6. Upstream Regulator Effects

Upstream regulator analysis was carried out with IPA software. This identifies any gene or small molecule which has been shown experimentally to affect gene expression as a potential upstream regulator. The regulator effects feature was used to explore the recGBM phenotype. The relationships between upstream regulators and downstream functions and diseases were plotted to identify drug targets. For each process, the significance (*p*-value) and directionality (z-score) between groups were visualized as a hierarchical heatmap. The size of each heatmap square was determined by the −log(*p*-value), and based on the z-score, orange and blue indicated processes activated in the recurrent and initial groups, respectively.

### 2.7. Gene Expression Connectivity Mapping

The gene signature of up-(53) and down-regulated DEGs (46) were investigated for identifying repurposing FDA-approved therapeutics using QUADrATiC software [21]. Using the gene signature, target compounds that would reverse the recurrent phenotype (i.e., negative connections) were identified. Results for all significant negative connections and those for brain cells and brain stem/progenitor cell types (i.e., NEU, NPC) were tabulated. NEU cells are normal terminally differentiated neuronal cells derived from iPS (induced pluripotent stem)-derived neural progenitor cells (NPC). Results were visualized as normalized contribution fraction (NCF) heatmaps, which indicate which genes are responsible for the identified connections.

## 3. Results

### 3.1. Samples, Data Quality Control, and Provisional Comparisons of Initial and recGBM

In brief, samples from eleven patients (eight male, three female) with an average age of 49 years of age at first diagnosis were analyzed. Molecular profiling confirmed all tumors were IDH1/2-wildtype, while four had MGMT promoter methylation as either low (5–10%; patients 6,11) or intermediate (10–25%; patients 7,10). Seven patient-matched pairs and four additional unmatched samples (*n* = 18) passed QC (Table 1). Initial and recurrent samples formed two groups in the PCA, indicating distinct transcriptional profiles (Figure S1). Stroma and immune scores were slightly higher in recurrent, but tumor purity was equivalent (*p*-value = 0.778; Table S1). Four of the seven patient-matched paired samples (57%) switched transcriptional subtypes after disease progression, while three remained the same, revealing no bias. RecGBM tumors were classified as mesenchymal (*n* = 3), proneural (*n* = 2), and classical (*n* = 3; Table S2; see Supplementary Materials). Further analysis was performed to compare transcriptional profiles of the patient-matched initial and recurrent GBM (*n* = 7).

### 3.2. Differential Gene Expression Analysis between Initial and recGBM

**Figure 1:**
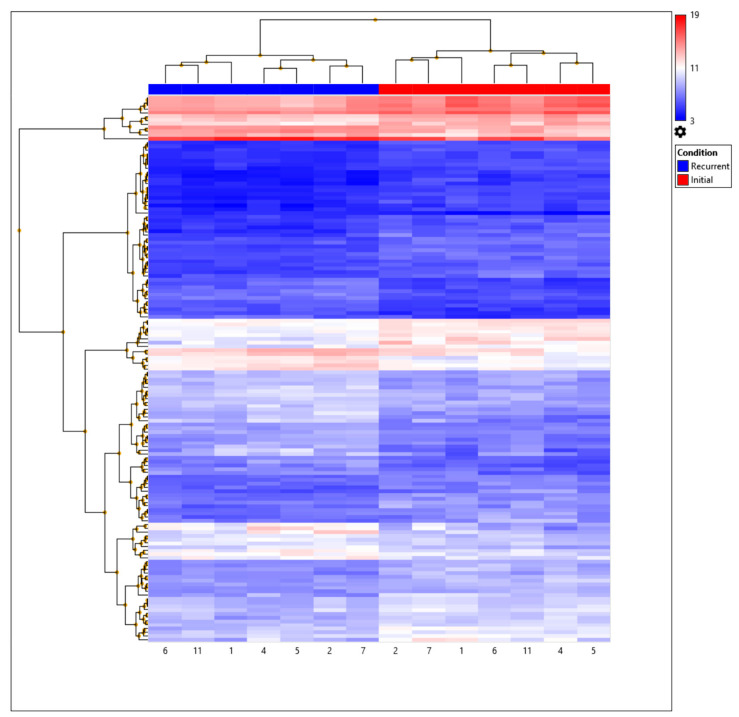
Hierarchical clustering of GBM IDH-wildtype samples based on the expression of the 147 differentially expressed probes or genes (DEGs). Expression (Log_2_) is displayed on a scale from high (red) to low (blue) values. Patient sample IDs of initial (red) and recurrent (blue) are displayed at the bottom of the heatmap.

Following filtering, 147 probes were identified as being differentially expressed (Table S3). Probes composed of coding (*n* = 14; 9.5%), non-coding (*n* = 54; 36.7%), small RNA (*n* = 8; 5.4%), precursor micro-RNA (*n* = 4; 2.7%), multiple complex (*n* = 46; 31.2%), and unassigned transcripts (*n* = 21; 14.2%). In total, 60 (40.8%) and 87 (59.2%) transcripts were up- and down-regulated, respectively, in recGBM (Figure S2). A greater proportion of the down-regulated transcripts were non-coding genes (49.4%) and small RNAs (9.2%), compared to up-regulated transcripts, which were mostly multiple complex genes (51.6%) and no small RNAs. Only 103 probes had gene symbols, and 30 of these were predicted ‘genes’ identified by ACEVIEW, but not described by GENCODE. A further eight probes lacked annotation and descriptions (e.g., RP11-318C24.1, AC007881.4, AL772161.2). Many of the remaining probes were related to unannotated tRNAs and some small RNAs etc. Thus, only 65 of the differentially expressed probes related to annotated functional genes. The top ten most significant DEGs were *ZEB1*, *RMST*, *GZMK*, *VSIG4*, *RPL30P7*, *HLA-DQA1*, *CPNE8*, *PER3*, *HLA-DRA*, and *CLEC7A*. According to expression (fold change), the most up-regulated DEGs included *HBB* (8.95), *HBA1* (8.63), and *HBA2* (7.22), and down-regulated ones included *CXCL8* (−4.84), *NAMPTP3* (−4.08), and *VSIG4* (−3.51). Initial and recurrent GBM stratified into distinct groups confirming that their transcriptional profiles differed significantly (Figure 1).

Expression data in four public cohorts were tested to validate DEGs. In total, 22 genes were found to also be differentially expressed between initial and recGBM in at least one other dataset (*p*-value < 0.05; Table S4). DEGs that were observed to be up-regulated in the Belfast and another cohort included *FPR3*, *SDC2*, *FCGR2B*, *GPNMB*, *CTSZ*, *AHR*, *HLA-DRA*, *CXCL12*, *LYZ*, *MEF2A*, *EIF1*, *RPL30*, and *EIF4A2*. DEGs that were observed to be down-regulated in the Belfast and another cohort included *BCAN*, *CNOT2*, *ABCG2*, and *GZMK*. Thus, the majority of genes (*n* = 17) showed the same directional regulation of expression as the Belfast cohort, with the exception of five genes (*SPOCK1*, *PTPRC*, *SCG3*, *CLEC7A*, and *DMXL2*).

The literature search revealed that 23 DEGs had known connections to GBM. Additional search results on the expression of these genes in recGBM returned studies for seven genes, which provided validation for a further two genes (*EGFR*, *CXCL8*), giving a total of 24 validated DEGs (Table S5). Patterns of expression for the five DEGs (*HLA-DRA*, *CXCL12*, *EGFR*, *BCAN*, and *GPNMB)* that were up-regulated in this study were corroborated. In contrast, the two genes, *SPOCK1* and *CXCL8,* significantly down-regulated in this study were previously reported to be up-regulated in recGBM (Table S5).

### 3.3. Functional Enrichment Analyses of the DEGs

#### 3.3.1. Gene Ontology Analysis

**Figure 2:**
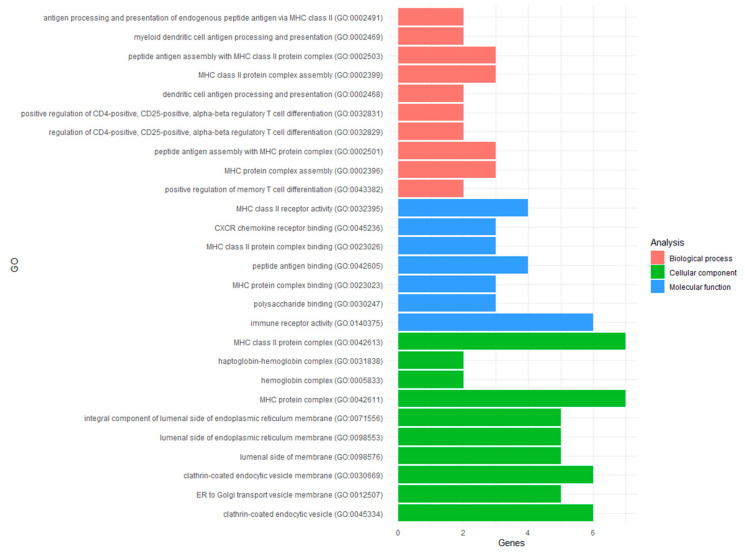
Results of the functional enrichment analyses of the DEGs identified in recGBM. Top ten GO terms for biological processes (red), molecular function (blue), and cellular components (green) are presented, including the number of genes overrepresented for each pathway.

In total, 180 significant GO terms for biological processes (N = 126), molecular function (N = 7), and cellular components (N = 47) were identified for the DEGs (Table S6). The top pathways had between two and seven DEGs overrepresented per pathway (Figure 2). Although this number of DEGs may seem low, it actually represented a 20 to >100 fold enrichment per pathway based on the background frequency of its genes expressed in the cohort (see Methods). Amongst the top results, ten GO terms related to the major histocompatibility complex (MHC). Specifically, DEGs were overrepresented in the MHC class II and protein complexes (GO:0042613; GO:0042611), including binding and receptor activity (GO:0023026; GO:0023023; GO:0032395) contributing to the biological process of antigen processing and the presentation of endogenous peptide antigen (GO:0002491). Furthermore, DEGS were overrepresented in myeloid dendritic cell antigen processing and presentation (GO:0002469) as well as the positive regulation of both memory (GO:0043382) and CD4+, CD25+, alpha-beta regulatory T-cell differentiation (GO:0032831; GO:0032829). Thus, DEGs reflect the transcriptional changes to recGBM related to the immune response.

#### 3.3.2. Canonical Pathway Analysis

**Figure 3:**
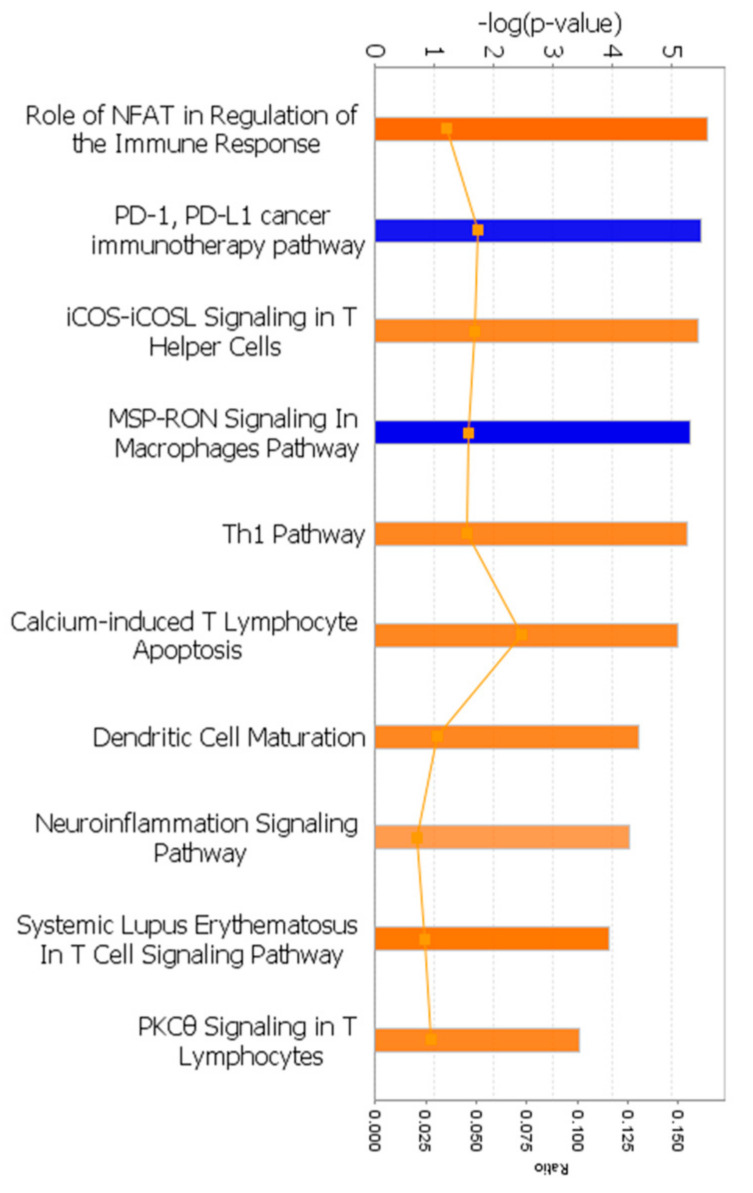
Canonical pathways activated (orange) or inhibited (blue) in the recGBM group. Each bar represents the pathway’s *p*-value on a negative logarithmic scale, such that the taller bars are more significant than the shorter bars. The ratio of the number of molecules present in the DEG list divided by the total number of molecules in the pathway is represented by the line graph (orange).

A total of 36 canonical pathways were significantly altered between initial and recGBM (Figure S3). The top three pathways were B-cell development, antigen presentation pathway, and allograft rejection signaling, implying significant changes in immune signaling. Pathways activated (orange) or inhibited (blue) in recGBM are presented in Figure 3. Eight pathways (z-score), all relating to the immune response, were activated in recGBM. These included NFAT in the regulation of immune response (2.449), iCOS-iCOSL signaling in T-helper cells (2.0), *Th1* pathway (2.0), calcium-induces T lymphocyte apoptosis (2.0), dendritic cell maturation (2.0), neuroinflammation signaling (1.633), systemic lupus erythematosus in T cell signaling (2.236), and *PKCθ* signaling in T lymphocytes (2.0). Inhibited pathways in recGBM included *PD-1*, *PD-L1* cancer immunotherapy (−2.0), and *MSP-RON* signaling in macrophages pathway (−2.236).

### 3.4. Upstream Regulator Effects

**Figure 4:**
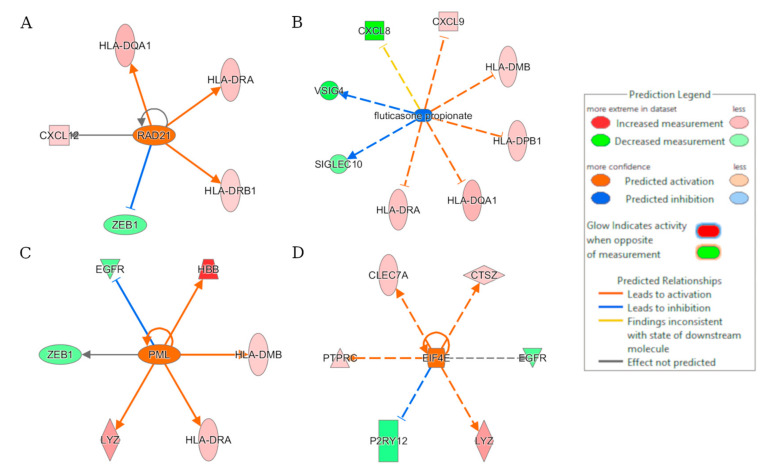
Results of the regulator effects analysis based on the DEGs. Predicted upstream regulators of the recGBM IDH-wildtype phenotype included (**A**) RAD21; (**B**) fluticasone propionate; (**C**) PML; and (**D**) EIF4E.

A total of 1279 upstream regulators were enriched in recGBM, linking them to activation or inhibition of gene expression. Among them, the genes (z-score), *RAD21* (2.0), *PML* (2.162), and *EIF4E* (2.236) were activated, and fluticasone propionate a corticosteroid that reduces inflammation (−2.315) was inhibited in recGBM (Figure 4). *RAD21* was the most significant, and its activation downregulates *CXCL12*, *HLA-DRB1*, and *HLA-DQA1* and up-regulates *ZEB1* resulting in the activation of the hematopoiesis of mononuclear leukocytes (Figure S4). Biological processes most significantly differentiating initial and recGBM in order were organismal injury and abnormalities (e.g., tumor growth), hematological system development and function (e.g., antigen, T-lymphocytes, mononuclear leucocytes), inflammatory response, and cancer and immune cell trafficking (see Figure S5; Supplementary Materials).

### 3.5. Gene Expression Connectivity Mapping

**Figure 5:**
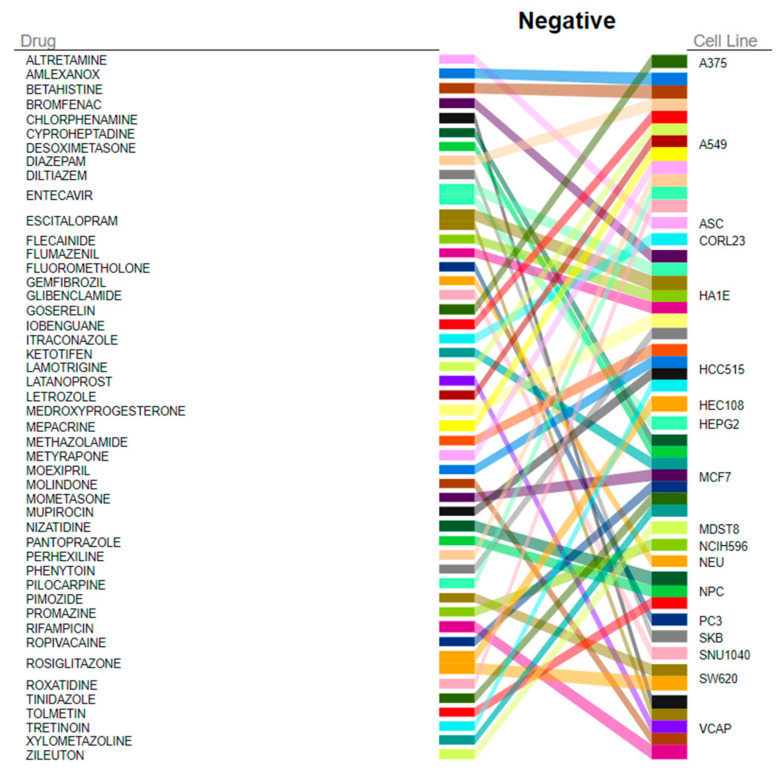
The top 30 FDA-approved target compounds identified from all cell lines by QUADrATiC software that could potentially reverse the recGBM phenotype.

**Figure 6:**
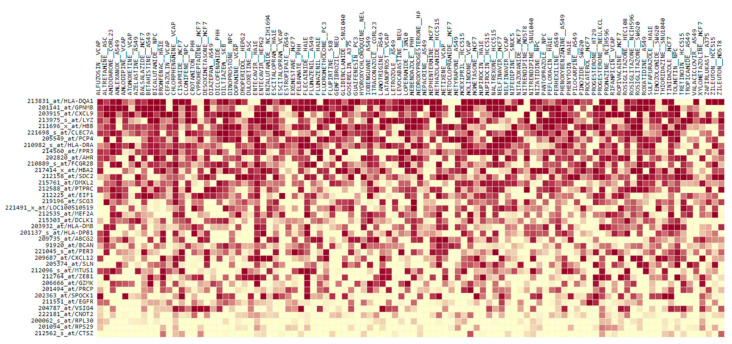
The normalized contribution fraction heatmap for the top 100 significant negative connections to target compounds identified for all cell lines identified by QUADrATiC software. Probes/genes have been sorted according to which DEGs in the signature are most affected by that drug. The DEG’s relative contribution is evident by their shading, which is on a scale from 1 (red) to −1.0149 (yellow).

The DEGs reflecting the transcriptional changes to recGBM were used in a connectivity mapping analysis to identify FDA-approved repurposing drugs that could potentially reverse the recGBM phenotype. In total, 114 significant negative connections, consisting of 98 unique compounds across 25 cell lines, were identified (Table S7). Overall, rosiglitazone was identified as the top and the fifth highest-ranking target compound identified from all cell lines (Figure 5). Amongst the other top five target compounds identified from all cell lines were escitalopram, rifampicin, and medroxyprogesterone. According to NCF, DEGs most affected by the identified drugs, in order, were *HLA-DQA1*, *GPNMB*, *CXCL9*, *LYZ*, *HBB,* and *CLEC7A* (Figure 6). Target compounds identified for the neuronally derived cell lines specifically, in order of significance, were nizatidine, pantoprazole, tolmetin, gemfibrozil, bicalutamide, progesterone, clomifene, hydroxychloroquine, dinoprostone, and levocabastine (Table 2). Dinoprostone was effective against ten different NPC cell lines, while hydroxychloroquine and gemfibrozil were both effective against six different NEU cell lines.

## 4. Discussion

There is a lack of treatment options for GBM once the standard of care (Stupp protocol) has been delivered to patients. Radiotherapy has limitations as a salvage therapy due to radiation toxicity and the potential selection of chemo-resistant tumor cell populations. More studies are revealing that recGBM tumors are both molecularly and transcriptionally distinct compared to the initial tumor following cytotoxic treatments. This necessitates the development of other therapeutic strategies that might be new to the tumor, and gathering transcriptional data from recGBM is crucial for this. In this study, initial and recGBM IDH-wildtype tumors were profiled using a whole transcriptome approach that included >540,000 transcripts and splice variants. From an initial cohort of 25 patients, data from only 11 patients (seven patient-matched pairs and four additional unmatched samples) was collected. Similar to other studies, this was technically challenging to achieve, as sample drop-out was high, impacting the sample size and statistical power of the study. Nevertheless, differential gene expression analysis of the patient-matched pairs revealed 147 differentially expressed probes. In total, 24 DEGs were validated using public cohorts and the literature. Up-regulated DEGs in recGBM were involved in angiogenesis (*SDC2*, *CXCL12*), immune-related processes (*HLA-DRA*, *FPR3*, *FCGR2B*, *AHR*, *LYZ*), tumorigenesis (*CTSZ*/*Cathepsin Z*) and metastasis (*GPNMB*), neuronal differentiation (*MEF2A*), and RNA binding (*EIF1*, *RPL30*, *EIF4A2*). Down-regulated DEGs in recGBM were involved in cell proliferation (*EGFR*) and possibly brain tumor cell growth (*BCAN*), deadenylation of mRNA, which is linked to neurodevelopmental disorders (*CNOT2*), multi-drug resistance (*ABCG2*), and immune-related processes (*GZMK*).

Angiogenesis genes, *SDC2* and *CXCL12,* were up-regulated in the recGBM group. *SDC2* is highly expressed in glioma microvasculature regulating angiogenesis [27]. For *CXCL12*, GBM potentially switches to a *CXCR4*-*CXCL12* angiogenic pathway from the well-known *VEGF*-*HIF1a* pathway [28]. RecGBM are frequently treated with bevacizumab, an anti-angiogenic drug that targets the *VEGF*-*VEGFR* axis with the aim of disrupting tumor angiogenesis; however, survival benefits with this monotherapy are negligible [29]. Immune-related genes, *HLA-DRA*, *FPR3*, *FCGR2B, AHR,* and *LYZ,* were also up-regulated in the recGBM group, with the exception of *GZMK,* which was down-regulated. In humans, MHC class II proteins are termed human leukocyte antigen (HLA) molecules, encoded by *HLA-DR*, *HLA-DP,* and *HLA-DQ* genes. *HLA-DR* expression is associated with tumor grade and prognosis in glioma [30,31]. Similarly, high expression of *FPR3* is associated with grade and *IDH* status in glioma [32]. A further study found that a gene signature of *FPR3,* along with two other genes, was prognostic for GBM [33]. *FCGR2B* was also prognostic for GBM singly and as part of an immune-related gene signature [34]. GBM patients with higher *FCGR2B* expression had shorter survival and were resistant to TMZ-nitrosoureas combination therapies [35]. *AHR* expression was also found to be associated with tumor grade and poor prognosis. High expression of *AHR* drives the expression of *CD39* in tumor-associated macrophages promoting CD8+ T-cell dysfunction [36]. In GBM, differential expression of *LYZ* and *PIK3AP1* alters the immune and tumor microenvironment leading to worse prognoses [37]. *GZMK (Granzyme K)* is a cytotoxic granule that promotes tumor death. Treatment of recGBM with neoadjuvant PD-1 checkpoint blockade improves survival outcomes by increasing anti-tumor T-cell responses, including *GZMK*; however, this is curtailed by additional T-cell checkpoints and other immunosuppressive pathways by the myeloid populations [38]. A better understanding of these processes could improve therapies. Other up-regulated DEGs were also involved in tumorigenesis (*CTSZ/Cathepsin Z*), metastasis (*GPNMB*), neuronal differentiation (*MEF2A*), and RNA binding (*EIF1*, *RPL30*, *EIF4A2*).

Down-regulated DEGs in recGBM included genes involved in cell proliferation (*EGFR*), possibly brain tumor cell growth (*BCAN*), deadenylation of mRNA, which is linked to neurodevelopmental disorders (*CNOT2*), and multi-drug resistance (*ABCG2*). The receptor tyrosine kinase *EGFR* is frequently amplified (∼57%) or mutated (∼11%) in primary GBM [4]. Overexpression of *EGFR* is often lower (44%) at GBM recurrence [39], corroborating our findings. *EGFR* inhibitors have yet to show clinical benefit against GBM; however, combinatorial therapies targeting both the *EGFR* and *STAT3* signaling pathways may have better therapeutic potential [40]. Single-cell transcriptomic profiling of dissociated GBM and peri-tumoral tissues observed neural stem cells in both, which included an *EGFR+ BCAN+* cell cluster, which may influence GBM recurrence [41]. *ABCG2* functions to efflux neurotoxic substances from the brain parenchyma to the bloodstream and may play a major role in multi-drug resistance [42]. Whilst *ABCG2* was significantly downregulated in recGBM, its expression is still very high compared to normal brain tissue, which could hinder therapy effectiveness [43].

Functional analyses revealed GO terms relating to an ‘MHC class II protein complex’ and ‘antigen processing and presentation’ as enriched in recGBM. MHC class II genes encode antigen-presenting peptides, which are key in initiating immune response [44]. These peptides are usually found on B-cells, macrophages, and dendritic cells. Down-regulation of MHC class II molecules has been associated with tumor cell invasion and immune evasion in glioma [45,46]. The enrichment of these genes in recGBM may indicate a change in the biological processes adopted by the tumor in response to therapy. Similarly, pathway analysis returned B-cell development, antigen presentation pathway, and allograft rejection signaling as the top three, further corroborating the observation of activated immune signaling in recGBM. The ‘Role of NFAT in the regulation of the immune response’ was the most significantly activated pathway. NFATs are calcium-dependent transcription factors that are required to be activated with the Fos-Jun complex for a productive immune response. High NFAT2 expression was associated with recGBM, the mesenchymal subtype, and poor survival, supporting our findings [47]. Inhibitors of the calcineurin-NFAT pathway suppressed proto-oncogenic pathways (hypoxia, glycolysis, PI3K/AKT/mTOR signaling axis) in vivo in GBM and, consequently, are being considered for therapies [48]. Additionally, two pathways inhibited in recGBM were ‘*PD-1*, *PD-L1* cancer immunotherapy’ and *‘SP-RON* signaling in the macrophages pathway’.

*RAD21* was identified as a potential upstream regulator of *CXCL12*, *HLA-DQA1*, *HLA-DRB1,* and *ZEB1* expression in recGBM. This may result in the downstream activation of ‘hematopoiesis of mononuclear leukocytes’, i.e., the formation of blood cells including both lymphocytes (B-cells, T-cells, NK cells) and monocytes (macrophages, dendritic cells). *RAD21* encodes a double-strand break repair protein; however, it also has roles as an upstream regulator. For example, the lncRNA, *MIR4697HG*, plays a role in gliomagenesis and progression [49]. Perhaps *RAD21* may be involved in the infiltration of antigen-presenting immune cells in recGBM. Several studies have been completed on the effects of immune cells, including mononuclear leukocytes, on GBM. Macrophages, originating in bone marrow, accumulate centrally in GBM tumors creating an immunosuppressive environment [50]. Assessment of the immune infiltration of GBM transcriptional subtypes found that the mesenchymal subtype had the highest microglia, macrophage, and lymphocyte infiltration, which could make it more susceptible to immunotherapy [51]. Results of the disease and function analysis highlighted the quantity of CD4+ T lymphocytes, differentiation of T lymphocytes and mononuclear leukocytes, and the proliferation of lymphocytes as potentially being increased in recGBM. This suggested a high infiltration of immune cells in recGBM while also highlighting increased inflammatory responses in the initial tumors. Inflammation, a natural immune response to infection, may be involved in gliomagenesis [52]. Therapies generating an injury response could be effective against GSCs that carry hallmarks of inflammation and may be responsible for gliomas via a neural wound response pathway [53]. In summary, increased activation of immune-related pathways was observed in recGBM. It should be noted that our GBM cohort was relatively young, with an average age of 49, and that these patients were “healthy” enough to be eligible for a second surgery. Thus, our results relate to a small and select patient cohort with a “resectable” recurrent tumor. It is not clear whether results might be transferrable to a “non-resectable” diffuse cohort. Nevertheless, our study suggests that recGBM may benefit from immunotherapy. To date, immunotherapies have not had significant clinical benefits for GBM patients. A lack of *HLA*-presented epitopes has been suggested as a limiting factor in the immunogenicity of GBM [54]. Our findings potentially suggest that *HLA-DQA1* could be targeted intratumorally in recGBM as part of CAR-T cell therapy, which might prove successful against these solid tumors [55].

These transcriptional changes to recGBM were utilized in a gene expression connectivity mapping analysis to identify FDA-approved compounds that could potentially be re-purposed in recGBM. The top-ranking compound across all cell lines was rosiglitazone, a synthetic agonist of the PPARγ nuclear hormone receptor [56]. It is also a member of the thiazolidinedione family of compounds (TZDs) that are synthetic ligands of the nuclear-receptor-peroxisome-proliferator-activated receptor gamma (PPARγ). PPARγ forms a heterodimer with retinoid-X-receptor for efficient ligand binding, after which the receptor-ligand complex binds DNA and induces signal trans-activation, regulating a spectrum of processes including glucose homeostasis, inflammation, and fatty acid metabolism [57]. Preliminary evidence from in vitro experiments by this group suggests that rosiglitazone in combination with radiation is effective against GBM cell proliferation [Al Rashid et al. in prep. unpublished results]. Compounds specifically identified for neuronal cell lines included nizatidine, pantoprazole, and tolmetin. Importantly, a compound structurally and pharmacologically related to the nonsteroidal anti-inflammatory drug tolmetin, ketorolac, and it is r-enantiomer, has been shown to inhibit small Rho GTPases (*Rac1*, *Cdc42*) and to reduce GBM infiltration in vitro [58]. A review of repurposing drugs to treat GBM only lists rosiglitazone, escitalopram, and hydroxychloroquine from the drugs we identified, and from those, only escitalopram has been tested in Phase II/II clinical trial (NCT02623231) [59]. All the other drugs we identified are novel suggestions for GBM and remain untested. High-content profiling of drugs is underway using a phenotypic approach on well-characterized GBM patient-derived cell lines by combining the Cell Painting assay with machine learning to classify drug mechanism of action [60]. Following successful in vivo and in vitro testing, the candidate compounds could be evaluated in future pre-clinical trials in combination with standard therapies, such as TMZ and bevacizumab, to target GBM and specifically the resistant GSC sub-populations in the initial tumor to prevent recurrence. Our translational bioinformatics pipeline provides an approach that could identify novel targets for cancer therapy. Finally, results from our whole transcriptome approach indicated that alterations in small non-coding RNAs, such as tRNAs, might have a role in disease progression in GBM IDH-wildtype. In other cancers, tRNAs are involved in apoptosis and tumorigenesis and have been successfully used as diagnostic biomarkers see [61]. Future work to characterize the non-coding portion of the transcriptome, including tRNAs, may prove useful to reveal additional mechanisms of disease regulation that could open new avenues for therapeutic interventions.

## Figures and Tables

**Table 1:** A summary of relevant clinical, treatment, molecular profiling, and diagnosis data for the GBM IDH-wildtype patients assessed. Time to relapse and overall survival are measured in days. The data file accession numbers for the initial and recurrent tumors are provided. TI = Technical Issue, TU = Technically unsatisfactory; * Isolated 19Q loss of heterozygosity, IHC = Immunohistochemistry, AMP/No AMP = Amplification.

Patient Identifier	P1	P2	P3	P4	P5	P6	P7	P8	P9	P10	P11
Initial	P1_I	P2_I		P4_I	P5_I	P6_I	P7_I	P8_I			P11_I
Recurrent	P1_R	P2_R	P3_R	P4_R	P5_R	P6_R	P7_R		P9_R	P10_R	P11_R
Sex	F	F	M	M	M	M	M	F	M	M	M
Age at diagnosis	56	55	35	54	33	64	45	55	36	61	45
Time to relapse	462	266	312	511	336	541	658	348	395	343	427
Overall survival	757	454	449	764	493	588	987	651	751	665	556
CCRT (weeks)	6	6	6	6	6	6	6	6	6	6	6
TMZ (cycles)	6	5	6	6	3	0	2	6	4	6	6
IDH1 (R132H) IHC	NEGATIVE	NEGATIVE	NEGATIVE	NEGATIVE	NEGATIVE	NEGATIVE	NEGATIVE	NEGATIVE	NEGATIVE	NEGATIVE	NOT PERFORMED
ATRX IHC	NEGATIVE (TI)	RETAINED	NEGATIVE (TI)	RETAINED	NEGATIVE (TI)	RETAINED	NEGATIVE (TI)	RETAINED	NEGATIVE (TI)	RETAINED	RETAINED
TERT	FAILED	NOT REPORTED	C250T MUTATION	FAILED	FAILED	NOT REPORTED	FAILED	FAILED	FAILED	C228T MUTATION	NOT PERFORMED
Histone H3F3A	NO MUTATION	NO MUTATION	NO MUTATION	NO MUTATION	NO MUTATION	NO MUTATION	NO MUTATION	NO MUTATION	NO MUTATION	NO MUTATION	NOT PERFORMED
IDH1/2 Seq	NO MUTATION	NO MUTATION	NO MUTATION	NO MUTATION	NO MUTATION	NO MUTATION	NO MUTATION	NO MUTATION	NO MUTATION	NO MUTATION	NO MUTATION
1p/19q co-deletion	RETAINED	RETAINED	RETAINED	RETAINED *	RETAINED	RETAINED	RETAINED *	RETAINED *	RETAINED	RETAINED	RETAINED
EGFR	NO AMP	NO AMP	NO AMP	AMPLIFICATION	NO AMP	AMPLIFICATION	NO AMP	AMPLIFICATION	AMPLIFICATION	NO AMP	AMP AND VIII MUTATION
MGMT promoter	NO/INSIGNIFICANT	NO/INSIGNIFICANT	NO/INSIGNIFICANT	NO/INSIGNIFICANT	NO/INSIGNIFICANT	LOW 5–10%	INTERMEDIATE 10–25%	NO/INSIGNIFICANT	NO/INSIGNIFICANT	INTERMEDIATE 10–25%	LOW 5–10%
Diagnosis	GBM, IDH-WT	GBM, IDH-WT	GBM, IDH-WT	GBM, IDH-WT	GBM, IDH-WT	GBM, IDH-WT	GBM, IDH-WT	GBM, IDH-WT	GBM, IDH-WT	GBM, IDH-WT	GBM, IDH-WT
Initial GEO Accession	GSM6508723	GSM6508725		GSM6508728	GSM6508730	GSM6508732	GSM6508734	GSM6508736			GSM6508739
Recurrent GEO Accession	GSM6508724	GSM6508726	GSM6508727	GSM6508729	GSM6508731	GSM6508733	GSM6508735		GSM6508737	GSM6508738	GSM6508740

**Table 2:** Significant negative connections to target compounds for neuronal cell lines identified by QUADrATiC software that could potentially reverse the recGBM phenotype.

FDA-Approved Drug	Description	No. of Profiles	Z-Score	Connection Score	*p*-Value	Cell Line *
nizatidine	Histamine H2-receptor antagonists	4	−5.376	−0.278	8.19 × 10^−8^	NPC
pantoprazole	Proton pump inhibitor	4	−4.458	−0.234	7.37 × 10^−6^	NPC
tolmetin	Nonsteroidal anti-inflammatory drug	3	−4.440	−0.301	9.66 × 10^−6^	NPC
bicalutamide	Anti-androgen drug	4	−4.250	−0.254	1.99 × 10^−5^	NPC
clomifene	Effective inhibitor of mutant IDH1	4	−4.076	−0.240	4.87 × 10^−5^	NPC
dinoprostone	Naturally occurring prostaglandin	10	−3.948	−0.145	7.40 × 10^−5^	NPC
levocabastine	H1 receptor antagonist	2	−3.929	−0.293	7.51 × 10^−5^	NEU
hydroxychloroquine	Regulates the immune system	6	−4.060	−0.189	4.98 × 10^−5^	NEU
progesterone	Endogenous steroid hormone	5	−4.189	−0.228	2.80 × 10^−5^	NEU.KCL
gemfibrozil	Lipid-lowering drug	6	−4.390	−0.193	1.01 × 10^−5^	NEU

* NPC: cells differentiated from induced pluripotent stem cells but not terminally differentiated. NEU: cells terminally differentiated to be neurons. NEU.KCL: cells terminally differentiated to be neurons and exposed to potassium chloride solution to activate neurons.

## Data Availability

The data is published in the Gene Expression Omnibus database under the data series accession number (GSE212067).

## Supplementary Materials

The following supporting information can be downloaded at: https://www.mdpi.com/article/10.3390/biomedicines11041219/s1. Figure S1: PCA plots generated for (A) all 18 GBM tumor samples profiled and (B) patient-matched initial and recurrent GBM samples only. Initial and recurrent tumors are labeled red and blue, respectively. Samples that did not pass the labeling control thresholds are labeled with a cube shape. One sample failed labeling QC (P11_I); however, as it did not appear as an outlier, it was maintained in the study; Figure S2: Results of the differential gene expression analysis between patient-matched initial and recurrent GBM. A: A volcano plot indicating the spread of up-regulated (red) and down-regulated DEGs were identified. B: Functional categories (%) assigned to each set of up-regulated and down-regulated DEGs; Figure S3: Results of the canonical pathways identified from the DEGs. A total of 36 canonical pathways were significantly altered between initial and recurrent tumors. Pathways activated (orange) or inhibited (blue) in the recurrent group are represented by bars with a positive or negative z-score, respectively. The ratio of the number of molecules present in the DEG list divided by the total number of molecules in the pathway is indicated in the line graph. Pathways having no activity pattern available (grey) meant that a z-score could not be calculated; Figure S4: Regulator effects analysis for *RAD21* revealed that its activation downregulates *CXCL12*, *HLA-DRB1*, *HLA-DQA1* and up-regulates *ZEB1* resulting in the activation of the hematopoiesis of mononuclear leukocytes; Figure S5: Hierarchical heatmap representing the biological processes most significantly differentiating the initial and recurrent groups based on the DEGs. Processes are sized according to their log(*p*-value) and colored according to z-score. Activation (orange) and inhibition (blue) in the recurrent group are represented by positive and negative z-scores of 1.5, respectively; Table S1: Comparison of the tumor composition results from ESTIMATE. Tumor purity did not significantly differ between initial (Mean = 0.7631 ± 0.0318 Stdev.) and recurrent (Mean = 0.7568 ± 0.0294 Stdev) samples (*p*-value = 0.778, F = 0.082, t = 0.437, df = 16); Table S2: Results of the transcriptional subtype assignment for all the initial and recurrent GBM IDH-wildtype samples. *p*-values were generated for each gene signature based on 1000 permutations of the gene set provided. For each sample, the lowest *p*-value was used to determine its subtype; Table S3: A list of the probes or genes that were identified as being differentially expressed between patient-matched initial and recurrent GBM IDH-wildtype; Table S4: Validation of the expression patterns of DEGs in other GBM cohorts. Results of the statistical comparison of expression of DEGs between initial and recurrent GBM in the four independent cohorts: CGGA (N = 75/85); Kwon et al. (N = 15); TCGA-GBM (N = 13); HF-MDA (N = 9). Trends for up (UP), down (DN), or opposite (O) gene expression regulation are indicated in comparison to the Belfast cohort, which is also listed. A column indicating whether the gene is validated in at least another cohort and showing the same expression trend is provided, and the test result is shaded. NS = Non-significant test result; NA = Not applicable because gene not available in the cohort for testing; Y = Yes; N = No; Table S5: Validation of the expression patterns of DEGs from the literature. Overview of the DEGs for which expression was reported for both initial and recurrent GBM in the literature; Table S6: Results of the GO analysis of DEGs related to Biological Process, Molecular Function, and Cellular Component; Table S7: Significant negative connections to target compounds that could reverse the recurrent GBM IDH-wildtype phenotype identified for all cell lines by QUADrATiC software. References [28,39,62,63,64,65,66] are cited in the Supplementary Files.
