# Supplementary material for: Transcriptional Profiling of a Patient-Matched Cohort of Glioblastoma (IDH-Wildtype) for Therapeutic Target and Repurposing Drug Identification
Source: Biomedicines. 2023 Apr 19;11(4):1219. doi: 10.3390/biomedicines11041219 (PMC10135924; doi:10.3390/biomedicines11041219)
Supplement: Supplementary file 1 [file biomedicines-11-01219-s001.zip › biomedicines-2345963-supplementary.pdf]

# SUPPLEMENTARY MATERIALS

## SUPPLEMENTARY METHODS

### RNA extraction of GBM tumors

Tissue sections underwent annotation by an experienced histopathologist to enable microdissection of tumor tissue from FFPE blocks. Total RNA extractions including DNAse treatment was performed on macrodissected tissue using the Roche High Pure RNA Paraffin Kit (Roche, Basel, Switzerland). RNA quantity and quality was assessed using a NanoDrop 1000 (Thermo Fisher Scientific, Wilmington, USA).

### Microarray profiling

Total RNA was amplified using the GeneChip WT Pico Reagent Kit (Thermo Fisher Scientific, Wilmington, USA). The protocol includes a reverse transcription step primer strategy to enhance amplification of partially degraded RNA from FFPE samples. The resulting amplified and biotinylated sense stranded DNA was hybridised to the Human Clariom$^{TM}$ D Human array (Thermo Fisher Scientific, Wilmington, USA). This array provides full coverage of the transcribed genome including all known coding and non-coding splice variants. Arrays were washed and stained using the FS-450 Fluidics Station before fluorescence intensity was measured using the GeneChip® Scanner 3000 7G with autoloader (Thermo Fisher Scientific, Wilmington, USA). Quality control (QC) measures were included (HeLa control RNA, poly-A bacterial RNA controls) to monitor target preparation and eukaryotic hybridization. All sample arrays were processed in a single run to remove potential batch effects.

### Data quality control and pre-processing

Transcriptome Analysis Console (TAC; ThermoFisher Scientific) software was used to carry out QC assessments and data summarisation prior to further analysis. This included checking the HeLa control, labelling controls, hybridisation controls, housekeeping genes and box plots of signal intensity. Signal Space Transformation combined with the Robust Multi-array Average algorithm were then used for data normalisation and summarisation. Principle Component Analysis (PCA) plots were generated for all samples and matched paired samples to examine the variance between samples, comparative groups and identify any potential outliers.

**Tumor Purity**

The ESTIMATE (Estimation of STromal and Immune cells in MAlignant Tumor tissues using Expression data) R package was used to further assess samples for tumor purity[25]. The algorithm uses gene expression data to estimate levels of infiltrating stromal and immune cells and estimates tumor purity by performing a single-sample Gene Set Enrichment Analysis (ssGSEA). As the Clariom$^{TM}$ D Human array includes many non-coding transcripts, tRNAs, and structural variants, the expression matrix was initially subsampled to 25,183 probes with unique gene symbols and this smaller dataset was used for the analysis. Tumor purity was compared between initial and recurrent GBM samples using a *t*-test in SPSS Statistics 28 (IBM).

**Transcriptional subtyping of initial and recurrent GBM IDH-wildtype**

Transcriptional subtyping was carried out on the initial and recurrent GBM samples using the classification method described by Wang *et al.*[23] which is based on the subtypes originally derived by Verhaak and involves performing ssGSEA. For each sample, the gene expression values are rank normalised and ordered and the empirical cumulative distribution functions

(ECDF) of the genes in the signature and the remaining genes are generated. The integral of the difference between the ECDFs is then calculated. This is similar to a gene set enrichment analysis (GSEA) but is based on absolute expression instead of differential expression. Since the scores for the three gene signatures (Proneural, Mesenchymal, Classic) are not directly comparable, a resampling procedure is implemented that allows the generation of random ssGSEA scores for each subtype allowing *P*-values to be generated for the raw ssGSEA scores of each sample. Re-sampling was performed 1,000 times and the resulting *P*-values were used as a basis to assign a subtype to each sample.

## SUPPLEMENTARY RESULTS

### Sample collection

A total of 15 GBM patients with patient-matched initial and recurrent samples were available for analysis, however, sample drop-out reduced this number. Six samples were deemed unsuitable for RNA extraction due to insufficient tumor tissue or degradation from necrosis. Following molecular profiling, a further three samples were excluded as they were re-classified as either IDH-mutant or oligodendrogliomas according to WHO guidelines[2]. All patients had their initial tumor resected prior to any treatment followed by a standard treatment protocol of radiotherapy and TMZ, except for patient 6 who received radiotherapy only (Table S1).

### Quality control

QC assessments revealed that array hybridization was successful for all samples. Similarly, all samples passed labelling control checks, with the exception of one sample which marginally failed (P11_I). Comparison to a set of positive controls revealed that all samples passed QC based on an Area under the curve (AUC) threshold of > 0.75. Examination of the signal

intensity of samples revealed no outliers. The PCA plot including all 18 samples revealed no clustering (Figure S1a). The sample which failed the labelling controls assessment (P11_I) did not appear as an outlier in either PCA. Given that this sample only narrowly failed one QC measure, it was maintained in the study.

**Tumor Purity**

The analysis was based on 4,054 genes, including 73 and 71 genes from the stroma and immune signatures, respectively. Whilst stroma and immune scores were slightly higher in recurrent GBM tumors (Table S2), tumor purity measures for initial (Mean = 0.7631 ± 0.0318 Stdev.) and recurrent (Mean=0.7568 ± 0.0294 Stdev) samples did not significantly differ ($P$ =0.778, F=.082, t=.437, df=16).

**Transcriptional Subtyping**

Transcriptional subtype classification was determined for all samples and compared between initial and recurrent tumors. Of the 18 GBM samples, six were assigned a mesenchymal subtype, four were assigned a proneural subtype and eight a classical subtype (Table S3). Thus, there appeared to be no bias amongst GBM samples towards a particular subtype. For the seven matched paired samples, four switched subtype (57%), while three remained the same after disease progression. It was interesting to note that both the initial and the recurrent groups comprised the same number of subtypes. These included four classified as mesenchymal, three as classical and one as proneural. Thus, subtype is unlikely to have been a confounding factor impacting upon results when comparing the patient matched initial and recurrent samples.

**Disease and biological function**

Within the category of Organismal Injury and Abnormalities, the results indicate that growth of malignant tumor (-log(p-value)=3.225, z-score=-1.104), growth of tumor (-log(p-value)=4.730, z-score=-1.039), inflammation of organ (-log(p-value)=8.294, zscore=-0.792), fibrosis (-log(p-value)=5.690, z-score=-1.934) and occlusion of artery (log(p-value)=4.447, z-score=-1.000) are activated in the initial group while inflammation of joint (-log(p-value)=9.788, z-score=0.561) , extracranial solid tumor (-log(pvalue)=4.652, z-score=0.339) and metastatic solid tumor (-log(p-value)=5.177, z-score=0.339) are activated in the recurrent group.

Within Haematological System Development and Function, the results indicate that the activation of antigen presenting cells (-log(p-value)=4.332, z-score=-1.131) is increased in the initial group while the quantity of CD4+ T-lymphocytes (-log(pvalue)=2.775, z-score=1.850), differentiation of T-lymphocytes (-log(p-value)=2.896, zscore=2.220) and mononuclear leukocytes (-log(p-value)=3.377, z-score=1.979), proliferation of lymphocytes (-log(p-value)=6.412, z-score=1.641) and haematopoiesis of mononuclear leukocytes (-log(p-value)=2.724, z-score=2.387) are all predicted to be upregulated in the recurrent group. Further analysis revealed the changes in immune response between the two groups had some overlap. This includes the upregulation of the activation of lymphocytes and leukocytes in the recurrent group and an increased inflammatory response in the initial group.

**Supplemental Tables**

Table S1. Comparison of the tumor composition results from ESTIMATE. Tumor purity did not significantly differ between initial (Mean = 0.7631 ± 0.0318 Stdev.) and recurrent (Mean=0.7568 ± 0.0294 Stdev) samples (*P*-value =0.778, F=.082, t=.437, df=16).

| Stage | | Stromal Score | Immune Score | ESTIMATE Score | Tumor Purity |
|---|---|---|---|---|---|
| Initial | Mean | 203.801 | 453.012 | 656.813 | 0.7631 |
| | N | 8 | 8 | 8 | 8 |
| | Std. Deviation | 201.023 | 137.237 | 332.638 | 0.0318 |
| Recurrent | Mean | 235.134 | 489.339 | 724.474 | 0.7568 |
| | N | 10 | 10 | 10 | 10 |
| | Std. Deviation | 172.790 | 154.479 | 303.493 | 0.0294 |
| Total | Mean | 221.208 | 473.194 | 694.402 | 0.7596 |
| | N | 18 | 18 | 18 | 18 |
| | Std. Deviation | 180.838 | 143.993 | 309.065 | 0.0297 |

Table S2. Results of the transcriptional subtype assignment for all the initial and recurrent GBM IDH-wildtype samples. *P*-values were generated for each gene signature based on 1,000 permutations of the gene set provided. For each sample, the lowest *P*-value was used to determine its subtype.

| Sample ID | Proneural *P*-value | Classical *P*-value | Mesenchymal *P*-value | GSEA subtype call |
|---|---|---|---|---|
| P1_I | 1 | 1 | 0.0495 | Mesenchymal |
| P1_R | 0.96 | 0.307 | 0.406 | Classical |
| P2_I | 0.545 | 0.446 | 0.0099 | Mesenchymal |
| P2_R | 0.99 | 0.871 | 0.0198 | Mesenchymal |
| P3_R | 0.0099 | 1 | 0.0198 | Proneural |
| P4_I | 1 | 0.228 | 0.99 | Classical |
| P4_R | 0.495 | 0.0099 | 0.0099 | Mesenchymal |
| P5_I | 0.0099 | 1 | 1 | Proneural |
| P5_R | 0.0198 | 0.683 | 0.0099 | Mesenchymal |
| P6_I | 0.98 | 0.0099 | 1 | Classical |
| P6_R | 1 | 0.228 | 1 | Classical |
| P7_I | 0.0396 | 0.941 | 0.0099 | Mesenchymal |
| P7_R | 0.0792 | 0.762 | 0.752 | Proneural |
| P8_I | 1 | 0.921 | 1 | Classical |
| P9_R | 0.822 | 1 | 1 | Proneural |
| P10_R | 0.0792 | 0.0099 | 1 | Classical |
| P11_I | 0.0198 | 0.0099 | 0.832 | Classical |
| P11_R | 0.0891 | 0.0297 | 0.832 | Classical |

Table S3. A list of the probes or genes that were identified as being differentially expressed between patient-matched initial and recurrent GBM IDH-wildtype.

Table S4. Validation of the expression patterns of DEGs in other GBM cohorts. Results of the statistical comparison of expression of DEGs between initial and recurrent GBM in the four independent cohorts: CGGA (N=75/85); Kwon et al. (N=15); TCGA-GBM (N=13); HF-MDA (N=9). Trends for UP, Down (DN) or opposite (O) gene expression regulation is indicated in comparison to the Belfast cohort which is also listed. A column indicating whether the gene is validated in at least another cohort and showing the same expression trend is provided and the test result is shaded. NS=Non-significant test result; NA= Not applicable because gene not available in cohort for testing; Y=Yes; N=No.

Table S5. Validation of the expression patterns of DEGs from the literature. Overview of the DEGs for which expression was reported for both initial and recurrent GBM in the literature.

| Gene | Title | Reference | UP/DN | Belfast |
|---|---|---|---|---|
| HLA-DRA | Immunophenotyping of Newly Diagnosed and Recurrent Glioblastoma Defines Distinct Immune Exhaustion Profiles in Peripheral and Tumor-infiltrating Lymphocytes | [62] | UP | UP |
| CXCL12 | Recurrence of glioblastoma after radio-chemotherapy is associated with an angiogenic switch to the CXCL12-CXCR4 pathway | [28] | UP | UP |
| EGFR | Expression of EGFR in Paired New and Recurrent Glioblastomas | [39] | DN | DN |
| BCAN | Effect of Concomitant Radiochemotherapy on Invasion Potential of Glioblastoma | [63] | DN | DN |
| GPNMB | Glioblastoma Recurrence Correlates With Increased APE1 and Polarization Toward an Immuno-Suppressive Microenvironment | [64] | UP | UP |
| SPOCK1 | SPOCK1 is upregulated in recurrent glioblastoma and contributes to metastasis and Temozolomide resistance | [65] | UP | DN |
| CXCL8 | Interleukin-8/CXCR2 signaling regulates therapy-induced plasticity and enhances tumorigenicity in glioblastoma | [66] | UP | DN |

Table S6. Results of the GO analysis of DEGs related to Biological Process, Molecular Function and Cellular Component.

Table S7. Significant negative connections to target compounds that could reverse the recurrent GBM IDH-wildtype phenotype identified for all cell lines by QUADrATiC software.

**Supplemental Figures**

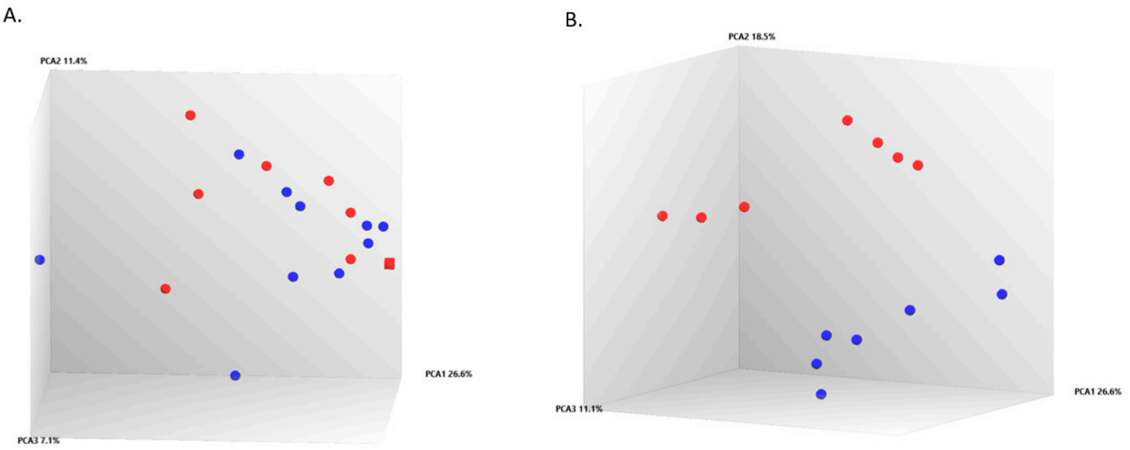


Figure S1. PCA plots generated for A) all 18 GBM tumor samples profiled and B) patient-matched initial and recurrent GBM samples only. Initial and recurrent tumors are labelled red and blue, respectively. Samples that did not pass the labelling control thresholds are labelled with a cube shape. One sample failed labelling QC (P11_I), however, as it did not appear as an outlier it was maintained in the study.

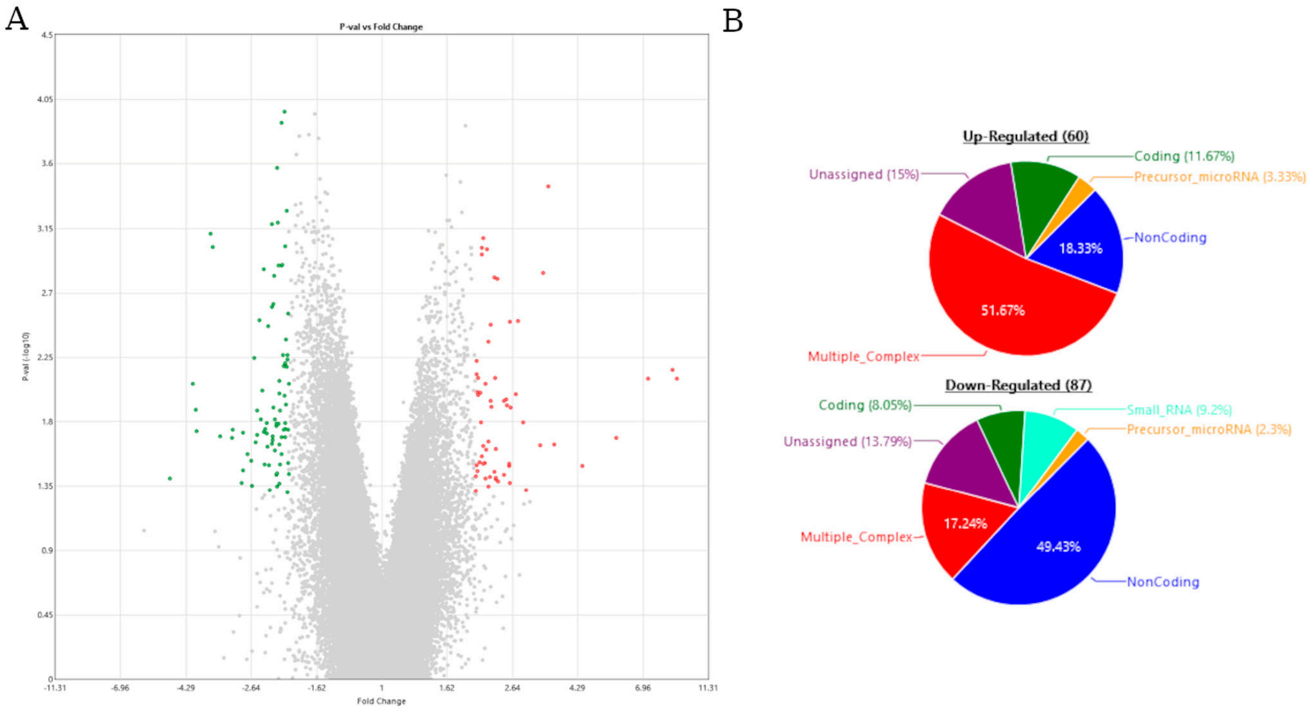


Figure S2. Results of the differential gene expression analysis between patient-matched initial and recurrent GBM. A: A volcano plot indicating the spread of up-regulated (red) and down-regulated DEGs identified. B: Functional categories (%) assigned to each set of up-regulated and down-regulated DEGs.

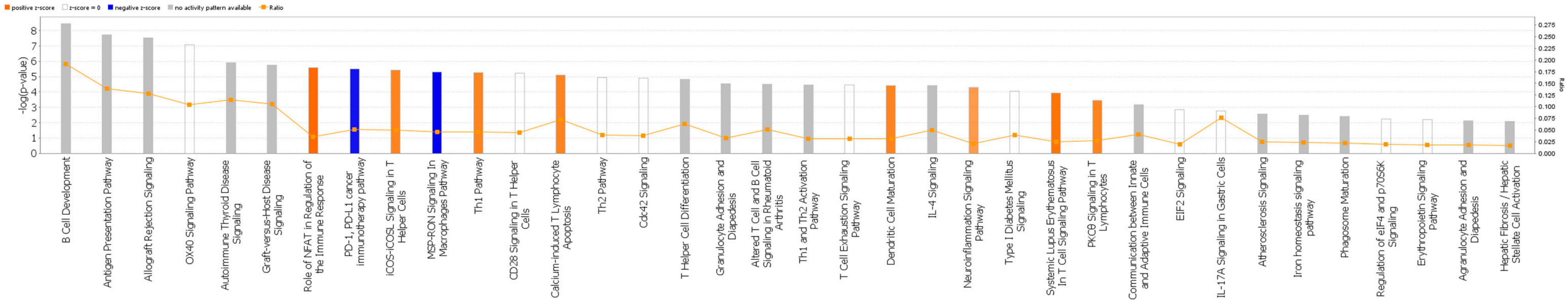


Figure S3. Results of the canonical pathways identified from the DEGs. A total of 36 canonical pathways were significantly altered between initial and recurrent tumors. Pathways activated (orange) or inhibited (blue) in the recurrent group are represented by bars with a positive or negative z-score, respectively. The ratio of the number of molecules present in the DEG list divided by the total number of molecules in the pathway is indicated with the line graph. Pathways having no activity pattern available (grey) meant that a z-score could not be calculated.

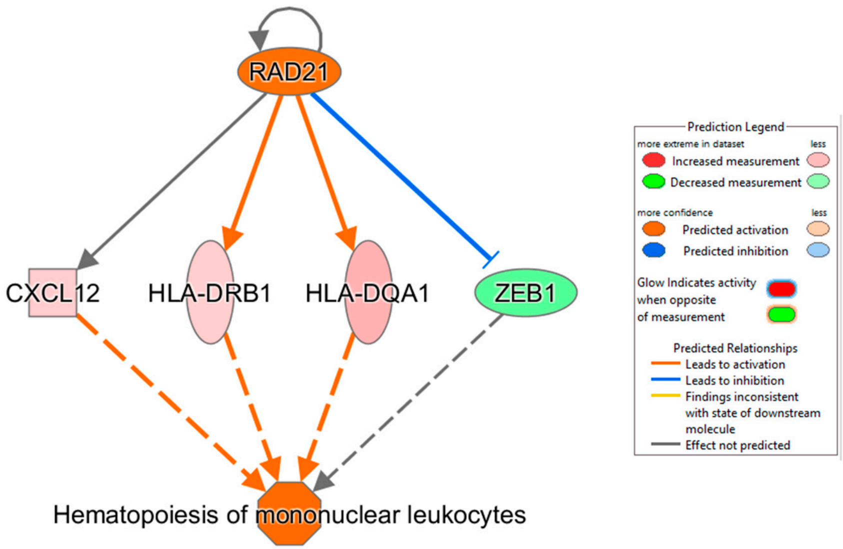


Figure S4. Regulator effects analysis for *RAD21* revealed that its activation downregulates *CXCL12*, *HLA-DRB1*, *HLA-DQA1* and upregulates *ZEB1* resulting in the activation of the haematopoiesis of mononuclear leukocytes.

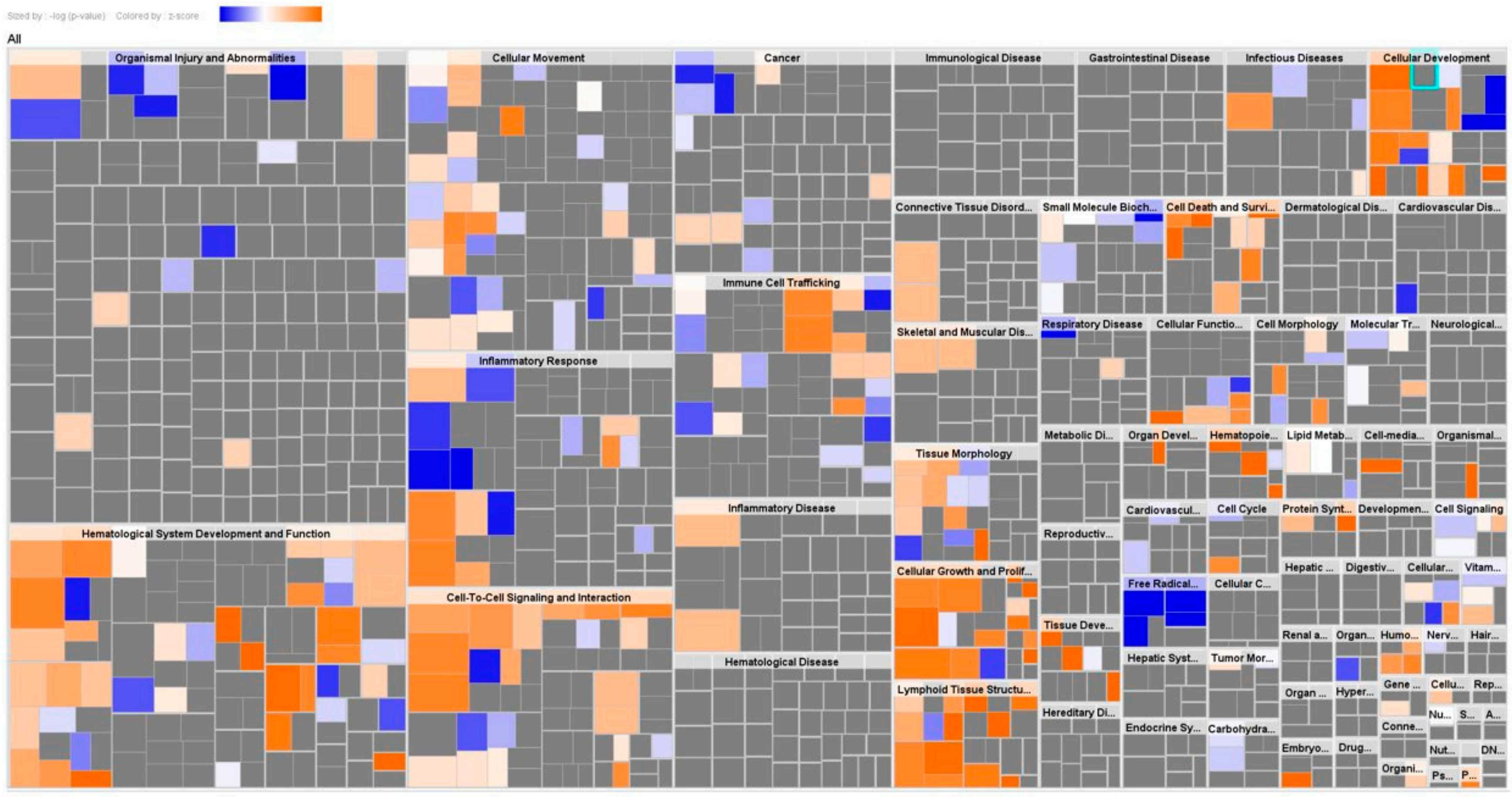


Figure S5. Hierarchical heatmap representing the biological processes most significantly differentiating the initial and recurrent groups based on the DEGs. Processes are sized according to their log(p-value) and coloured according to z-score. Activation (orange) and inhibition (blue) in the recurrent group are represented by positive and negative z-scores of 1.5, respectively.
